# 384. Factors Potentiating the Risk of Linezolid-induced Thrombocytopenia in Patients without Haemato-oncologic Diseases: A Case-control Study

**DOI:** 10.1093/ofid/ofad500.454

**Published:** 2023-11-27

**Authors:** Abrar K Thabit, Arwa A Alghamdi, Afnan K Alsaeed, Nesreen M Magbool, Ahmad J Mahrous, Alya Alrowaili, Yazed S Alsowaida, Ziyad K Albakistani, Basem O Albangali, Anas M Alghumuy, Sara A Youssef, Reem M Alodayli

**Affiliations:** King Abdulaziz University, Jeddah, Makkah, Saudi Arabia; King Abdulaziz University, Jeddah, Makkah, Saudi Arabia; King Abdulaziz University, Jeddah, Makkah, Saudi Arabia; King Abdulaziz University, Jeddah, Makkah, Saudi Arabia; Umm Al Qura University, Jeddah, Makkah, Saudi Arabia; King Fahad Medical City, Riyadh, Ar Riyad, Saudi Arabia; Ha’il University, Hail, Ha'il, Saudi Arabia; Umm Al-Qura University, Makkah, Makkah, Saudi Arabia; Umm Al-Qura University, Makkah, Makkah, Saudi Arabia; Umm Al-Qura University, Makkah, Makkah, Saudi Arabia; Saudi German Hospital, Hail, Ha'il, Saudi Arabia; Saudi German Hospital, Hail, Ha'il, Saudi Arabia

## Abstract

**Background:**

Thrombocytopenia is a major adverse effect of linezolid due to myelosuppression. Many studies assessed factors associated with this effect, but most included patients with haemato-oncologic diseases, which may confound the assessment of thrombocytopenia. Therefore, this study aimed to investigate risk factors for linezolid-induced thrombocytopenia in patients without haemato-oncologic diseases.

**Methods:**

This was a multicenter retrospective case-control study of adults treated with linezolid 600 mg twice daily for ≥ 3 days. Cases were the patients who developed thrombocytopenia while controls are those who didn't develop it. Exclusion criteria were haemato-oncologic diseases, active COVID-19, active dengue fever, baseline platelet count < 100 × 10^3^/mm^3^, concurrent therapy with trimethoprim/sulfamethoxazole or valproic acid, platelet transfusion within 7 days before linezolid therapy, and insufficient platelets data. Various factors were assessed for potential association with linezolid-induced thrombocytopenia (defined as drop in platelet count below 100 × 10^3^/mm^3^).

**Results:**

142 patients were included, 31 cases and 111 controls. Patients in both groups had a median age of 62 years and received linezolid for a median of 8 days. Platelet counts significantly dropped in the cases (Figure 1). Figure 2 shows the trend of change in platelet count (the white square indicates the median duration to thrombocytopenia, 8 days). In the univariate analysis, serum creatinine, creatinine clearance, baseline hemoglobin, baseline platelet count, and bacteremia were significantly associated with thrombocytopenia. However, after adjusting for confounders in the multivariate analysis, only low baseline hemoglobin and bacteremia were associated with thrombocytopenia (adjOR = 0.71; 95% CI 0.53-0.95; *P* = 0.022 and adjOR = 17.02; 95% CI 1.15-251-71; *P* = 0.039, respectively). Among the cases, those who had a baseline platelet count of < 200 × 10^3^/mm^3^ developed thrombocytopenia faster as early as 3 days (log-rank *P* = 0.002; figure 3).

**Figure 1**

**Figure:**
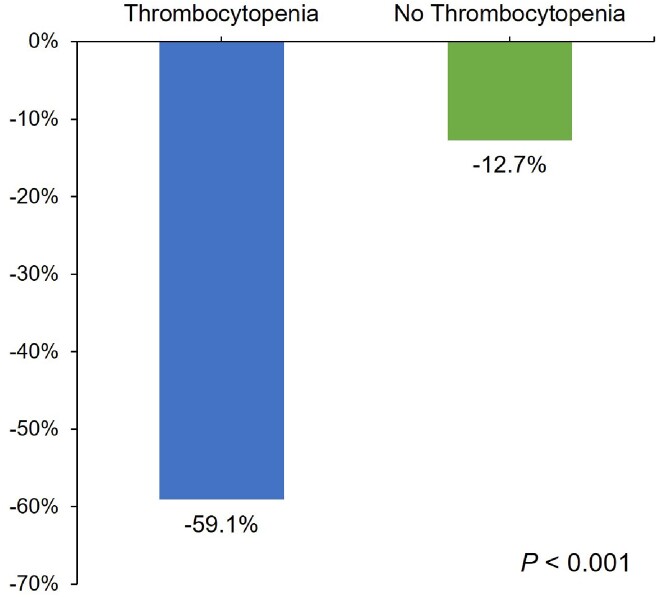

Rate of change in platelet count between the cases (thrombocytopenia) and controls (no thrombocytopenia)

**Figure 2**

**Figure:**
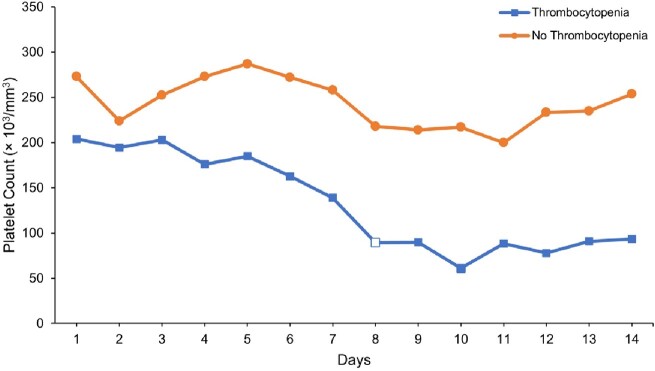

Trend of platelet counts over 14 days in the cases (thrombocytopenia) and controls (no thrombocytopenia)

**Figure 3**

**Figure:**
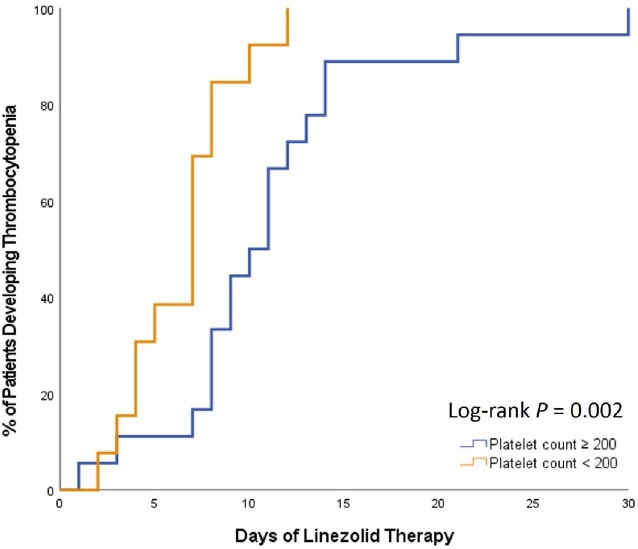

Kaplan-Meier curve for the development of thrombocytopenia depending on baseline platelet count

**Conclusion:**

Patients without haemato-oncologic diseases are at risk of linezolid-induced thrombocytopenia. especially those with bacteremia and low hemoglobin level. Patients with low baseline platelet count should be monitored closely early in therapy.

**Disclosures:**

**All Authors**: No reported disclosures

